# Importance of Illness Acceptance Among Other Factors Affecting Quality of Life in Acromegaly

**DOI:** 10.3389/fendo.2019.00899

**Published:** 2020-01-14

**Authors:** Aleksandra Jawiarczyk-Przybyłowska, Dorota Szcześniak, Marta Ciułkowicz, Marek Bolanowski, Joanna Rymaszewska

**Affiliations:** ^1^Department of Endocrinology, Diabetes and Isotope Therapy, Wroclaw Medical University, Wroclaw, Poland; ^2^Department of Psychiatry, Wroclaw Medical University, Wroclaw, Poland

**Keywords:** acromegaly, quality of life, disease activity, acceptance of illness, psychopathology

## Abstract

**Introduction:** The aim of this study was to analyze psychological factors of patients with acromegaly and assessment of their relationship with the quality of life (QoL) in the context of the control of the disease.

**Materials and methods:** A total sample of 50 patients (62% of females; mean age = 51.66 ± 14.5) with acromegaly underwent a comparative, cross-sectional cohort assessment including the QoL (AcroQoL, WHOQoL-BREF), psychiatric morbidity (GHQ-28), the acceptance of illness (AIS) as well as influence of treatment, comorbidities and symptoms in the relation of disease activity. Acromegaly group was divided in two subgroups: patients with uncontrolled acromegaly (UA, *n* = 28) and patients with controlled acromegaly (CA, *n* = 22).

**Results:** The acromegaly groups did not differ in health-related QoL measured with AcroQoL and WHOQoL questionnaires. However, obtained results showed QoL impairments in all subscales and the study participants had decreased scores compared to reference values. The interaction of the relationship between the AIS and disease activity as well as the prevalence of all psychopathological symptoms and disease activity were tested and the statistically significantly differences in the context of QoL in AcroQoL questionnaires and its domains were observed in relation to the course of the disease. No difference in acromegaly symptoms as well as in number of comorbidities were found between CA and UA but these two parameters affected the results QoL scores in AcroQol questionnaires and their domains, regardless the disease activity. Similarly, the prevalence of psychopathological symptoms (GHQ-28) contributed the level of acceptance of the disease, regardless the disease activity. The strongest predictors of QoL were related to the level of illness acceptance (*p* = 0.01) as well as serum growth hormone concentration.

**Conclusion:** Minding people with UA, the control of biochemical factors seemed to be more important for the QoL perception, while among CA, psychological variables such as AIS are observed to play a fundamental role in QoL. Moreover, inclusion of patient's acceptance of the illness into clinical routine would promote holistic, patient-centered care and empower doctor-patient partnership where patients' expectations and perceptions are constantly tracked. Obtaining biochemical control should not be considered as the only measure of treatment success.

## Introduction

Acromegaly is a rare disease, predominantly associated with growth hormone-secreting pituitary adenoma. The total prevalence ranges between 2.8 and 13.7 cases per 100,000 people (1). Increased level of growth hormone (GH) results in extensive peripheral secretion of insulin-like growth factor-1 (IGF-1), both of which in a long term are in charge of unique set of problems such as alterations in body and facial appearance and defect of major organs. Effective treatment reduces the severity of symptoms resulting from the GH excess, such as increased sweating or soft tissue swelling. On the other hand, some changes are permanent, for example changes in facial appearance or destruction of bones and cartilages (2). It is estimated that the treatment of 50% of the patients is considered to be suboptimal which means they deal with consequences of long-term uncontrolled course of the disease, some of the symptoms are at least reversible with successful treatment (3). What is more, the reduction of GH to the desirable level and normalization of IGF-1 (defined as GH concentration < 2.5 ng/ml and IGF-1 within normal range) not always guarantee disappearance of accompanying symptoms such as headache, which significantly diminish quality of life (QoL) (4). Managing satisfying QoL is, in turn, listed guidelines among normalization of hormone levels as a tool to assess the effectiveness of treatment (5, 6). Additionally, in the light of multisystem-associated morbidities, optimal disease management seems to be crucial to prevent major side effects that may in turn lead to premature morbidity (3, 7). In the study by Liu et al. almost half of the patients with acromegaly presented with 5 or more comorbidities, the most common of which was depression at 56.6%. Moreover, 83.6% reported that symptoms interfere with their daily life and work (8). What is interesting, the appearance seems to be the most affected dimension while assessing QoL (9). According to a Dutch study, even after a long-term biochemical remission, the self-consciousness about appearance occurs. The face was indicated as the most prominent source of this anxiety (10). Complex system of manifestations that influence each other and may persist or even worsen regardless treatment requires continuous multi-specialty approach. The necessary attention should be given to provide psychological and psychiatric support with respect of the adequate assessment of the psychosocial and emotional consequences of the illness. It is also worth to noting that delay in diagnosis has been associated with psychosocial impairment, including depression, body image distortion, and social withdrawal (11, 12). As the course of the disease is the rather insidious chronic and slowly progressing, many patients experience a prolonged diagnostic process, to the extent of 7–10 years which strongly negatively affects receiving appropriate interventions in a timely manner (1, 2).

The effect of GH and IGF-1 normalization on patient's self-reported well-being is still questionable suggests though that QoL in patients with acromegaly is a multifactorial issue requiring individualized approach and points out (13–16). It is also known that other factors may be important, such as duration of the disease, age, disease activity, gender which playing the main role and correlates with QoL (4, 17, 18). All things considered, literature on the factors modulating QoL of patients with acromegaly suggests that further research is still required to provide them with patient-tailored assessment and therapeutic interventions.

The aim of this study was to analyze the psychological factors of patients living with acromegaly and to assess their relationship with the perceived QoL in the context of the control of the course of the disease.

## Materials and Methods

A comparative, cross-sectional cohort study was conducted at the Department of Endocrinology, Diabetes and Isotope Therapy, Wroclaw Medical University during the years 2012–2015.

Fifty participants were enrolled in the study. The study was approved by the local ethical committee. All the participants signed an informed consent before entering the study. The sample consisted of group of acromegaly patients (A), *n* = 50. Acromegaly group was divided in two subgroups: patients with uncontrolled acromegaly (UA, *n* = 28) and those with controlled disease (CA, *n* = 22). Demographic characteristics and medical history, which included the history of treatment (surgery, radiotherapy, pharmacological treatment), disease duration, and pituitary function were assessed in all patients. The GH concentrations were measured by a chemiluminescent immunometric method (Immulite 2000, Siemens, USA or Germany). Serum IGF-1 level was assessed by radioimmunologic assay using an IGF-I-D-RIT-CT kit (BioSource S.A., Nivelles, Belgium), normal range: according to the sex and age. The levels of GH and IGF-1 were measured during the recruitment for current analysis and based on which patients were qualified to the appropriate groups (UA vs. CA). Acromegaly was confirmed by nadir serum GH during an oral glucose tolerance test (OGTT) > 0.4 ng/ml and elevated IGF-1 for age and gender. Age and gender normalized levels of IGF-1, associated with nadir serum GH during OGTT < 0.4 ng/ml after surgery or random GH < 1.0 ng/ml when treated with somatostatin analogs were taken as criterion of cure or good disease control (19).

### Instruments

The following instruments were used in the patients' groups:

1. A sociodemographic profile sheet.
2. Clinical profile sheet consisted of the following details: disease onset and duration, delay of diagnosis, first symptoms, treatment, co-morbidities, and hormonal and biochemical measurement.
3. The 26-item Polish version of the WHO Quality of Life Scale-BREF (WHOQoL-BREF). It profiles the subjective evaluation of the QoL in the past 2 weeks within 4 domains: physical health (PhyHealth), psychological health (PsyHealth), social relationship (SocRel), and environment (Enviro).
4. The AcroQoL-Polish version for assessing the QoL in patients with acromegaly was used after obtaining permission by the authors. It consists of 22 items of QoL (AcroTotal). The questionnaire is divided into 2 scales that measure physical (AcroPhy) and psychological aspects (AcroPsy). The psychological scale is further divided into 2 subscales, the appearance (AcroApp) and personal relationships (AcroRel).
5. The 28-item version of the General Health Questionnaire (20) in Polish adaptation was used (21). The scale allows to measure general health status and its four components (each consists of 7 questions): A—GHQ-somatic symptoms, B—GHQ-anxiety and insomnia, C—GHQ-social dysfunction, and D—GHQ-severe depression. Higher scores indicate a greater probability of psychiatric distress.
6. The standardized Acceptance of Illness Scale (AIS) in Polish adaptation (22) consists of 8 questions describing the consequences of poor health condition. Higher scores indicate a better acceptance of the illness.

### Statistical Analysis

The analysis of differences between the group with controlled and uncontrolled acromegaly was performed using Fisher's test for qualitative variables or the Mann-Whitney test for quantitative variables. The values of variables are presented by specifying the mean value ± standard deviation. Analysis of the impact of quantitative variables (for example GHQ-28, AIS, etc.) including the division into groups of acromegaly was performed using a multifactorial regression analysis with an element of interaction. Similarly, differences in the QoL when divided by the acromegaly group and qualitative variables (for example surgery etc.) were examined using two-way ANOVA analysis. The independent impact of variables on QoL was performed using multifactorial regression analysis. As statistically significant, *p* value on the level below < 0.5 was used. The analysis was performed in the R for windows software (version 3.6.1) (23).

## Results

A total of 50 patients with acromegaly were assessed: 31 females and 19 males, mean age was 51.66 ± 14.5 years, with a mean disease duration of 8.4 ± 8.77 years. A group of 36 patients underwent pituitary adenoma surgery, 6 of them had additional radiotherapy. Only 10 patients were successfully treated with operation and required no additional medical interventions. Twenty six patients were treated with somatostatin analogs (SA). Among the whole study group, 22 patients were qualified to CA group (10 with successfully treated with operation and 12 well-controlled with SA) and 28 patients were certified as UA (14 with newly recognized acromegaly and 14 with non-successfully treatment such as operation, radiotherapy, and actually treatment with SA). The sociodemographic profiles in terms of gender, education, place of residence and marital status did not vary significantly between groups. Statistical significance was obtained regarding age and duration of the disease. Controlled acromegaly group was older compared to UA group (*p* = 0.003). Duration of the disease since time of diagnosis was higher in UA compared to CA (*p* = 0.0015). The levels of IGF-1 and GH were statistically significantly higher in patients with UA compared to CA which confirmed correct division of groups. The study groups characteristics was presented in Table 1.

**Table 1:** Demographic characteristics of the acromegaly groups.

	**Acromegaly group (A) *N* = 50**	**Controlled acromegaly subgroup (CA), *N* = 22**	**Uncontrolled acromegaly subgroup (UA) *N* = 28**	***p*-value**
Gender (females) *n* (%)	31 (62%)	15 (68.2%)	16 (57.1%)	
Age (mean years ± SD)	51.66 ± 14.5	57.91 ± 14.11	46.75 ± 13.10	CA vs. UA *p* = 0.003
Education *n* (%)				
Basic	4 (8.1%)	3 (13.6%)	1 (3.5%)	
Medium	20 (40.8%)	12 (54.5%)	8 (29.6%)	
High	14 (28.6%)	3 (13.7%)	11 (40.7%)	
Vocational	11 (22%)	4 (18.2%)	7 (25.9%)	
Unknown	1 (0.5%)		1	
Locality of living *n* (%)				
City	35 (70%)	13 (59.09%)	22 (78.57%)	
Country	15 (30%)	9 (40.91%)	6 (21.43%)	
Time since diagnosis (in years)	8.4 ± 8.77	12.13 ± 8.7	5.20 ± 7.66	CA vs. UA *p* = 0.0015
Marital status *n* (%)				
Married	34 (69.39%)	18 (81.82%)	16 (59.26%)	
Single	15 (30.61%)	4 (18.18%)	11 (40.74%)	
Occupation *n* (%)				
Employed	21 (43.7%)	7 (31.81%)	14 (53.84%)	
Unemployed	1 (2.08%)	0	1 (3.84%)	
Sickness benefit	12 (25%)	5 (22.72%)	7 (26.92%)	
Retirement	14 (29.16%)	10 (45.45%)	4 (15.38%)	
IGF-1 (ng/ml) (mean ± SD)	434.5 ± 291.6	217 ± 137.5	618.6 ± 259.0	CA vs. UA *p* = 0.000
GH (ng/ml) (mean ± SD)	6.32 ± 8.81	1.53 ± 1.28	9.86 ± 10.28	CA vs. UA *p* = 0.000

The comparison of the QoL among CA and UA subgroup is presented in Table 2 (WHOQol-BREF and AcroQoL). In WHOQoL-BREF scale study participants with uncontrolled course of disease showed slightly worse scores in psychical, psychological, and environmental domain and better scores in social relation but the difference was not statistically significant. In the same scale the whole study group obtained the lowest scores in social relation (a = 11.64 ± 2.3) and the highest in environmental domain (a = 29.68 ± 4.68). The acromegaly groups (controlled and uncontrolled) did not differ in health-related QoL measured with AcroQoL questionnaires. However, obtained results showed QoL impairments in all subscale, especially in appearance among the total study sample. The study participants had decreased AcroQoL scores compared to reference values.

**Table 2:** Quality of life and psychopathological status of acromegaly group.

	**A (*n* = 50) Mean ± SD**	**CA (*n* = 22) Mean ± SD**	**UA (*n* = 28) Mean ± SD**	***p*-value**
**WHOQoL-BREF**				
Physical health	55.28 ± 9.6	55.32 ± 11.63	55.25 ± 7.87	0.93
Psychological health	63.98 ± 10.85	66.23 ± 10.76	62.21 ± 10.78	0.25
Social relation	69.4 ± 17.84	65.64 ± 20.4	72.36 ± 15.64	0.28
Environmental	69 ± 14.7	71.23 ± 16.26	68.71 ± 13.56	0.70
AcroQoL total (min 22–max 110)	71.92 ± 14.11	70.95 ± 18.39	72.68 ± 9.85	0.80
Physical dimension (min 8–max 40)	25.22 ± 6.39	24.41 ± 8.23	25.86 ± 4.54	1
Psychological dimension (min 14–max 70)	46.6 ± 8.74	46.55 ± 11.09	46.64 ± 6.53	0.57
Appearance (min 7–max 35)	19.68 ± 5.57	20.64 ± 6.77	18.93 ± 4.40	0.32
Personal relationship (min 7–max 35)	26.96 ± 4.63	25.91 ± 5.98	27.79 ± 3.08	0.49
GHO-28 (total)	5.5 ± 5.81	5.5 ± 6.37	5.5 ± 5.45	0.87
A—somatic symptoms	1.6 ± 2.1	1.68 ± 2.25	1.53 ± 2.02	0.93
B—anxiety and insomnia	2.16 ± 2.1	2.27 ± 2.22	2.10 ± 1.89	0.95
C—social dysfunction	1.10 ± 1.63	1.27 ± 1.77	1.0 ± 1.54	0.84
D—severe depression	0.62 ± 1.2	0.36 ± 0.65	0.82 ± 1.46	0.50

There were no significant differences in prevalence of psychopathological symptoms among CA and UA groups, but the worst disturbances were observed in anxiety and insomnia scale in the whole group as well as in both study subgroups separately (Table 2).

The level of AIS was similar in UA and CA subgroups and indicated moderate AIS. There was no significant difference as long as acceptance of acromegaly is considered in controlled and uncontrolled group.

Moreover, the interaction of the relationship between the AIS and disease activity was tested and a statistically significantly difference in the context of QoL in AcroQoL questionnaires and its domains was observed in relation to the course of the disease. In the both groups of participants the higher the level of acceptance of the disease, the higher scores in AcroQoL was observed, but in uncontrolled group the size effect was significantly smaller (*r* = 0.885 vs. *r* = 0.432, respectively). Similar relationships were observed in three domains of AcroQol. In both groups, the higher acceptance of the disease, the higher scores in Physical Dimension, Psychological Dimension, and Personal Relationship were observed but the size effect was significantly smaller in uncontrolled group (*r* = 0.857 vs. *r* = 0.482; *r* = 0.831 vs. *r* = 0.367; *r* = 0.874 vs. *r* = 0.139, respectively). Moreover, the level of acceptance of the disease affected perception of appearance, regardless the disease activity (CAG and UAG) (*r* = 0.58 vs. *r* = 0.41, respectively) (Table 3).

**Table 3:** Linear mixed model analysis—interaction effect between WHOQoL-BREF scores and AcroQoL with AIS, comorbidities, and number of symptoms in study groups.

**Tool**	**Interaction description**	**Estimate**	**Std error**	***p*-value**
WHOQoL D3	UA AIS UA × AIS	67.90 1.60 −1.93	17.94 0.36 0.54	0.00^*^ 0.00^*^ 0.00^*^
WHOQoL D4	UA AIS UA × AIS	21.21 0.98 −0.76	16.39 0.33 0.39	0.20 0.00^*^ 0.13
AcroQoL All	UA AIS UA × AIS	39.92 1.82 −1.2	10.58 0.21 0.32	0.00^*^ 0.00^*^ 0.00^*^
Physical dimension	UA AIS UA × AIS	16.48 0.79 −0.48	4.95 0.10 0.15	0.00^*^ 0.00^*^ 0.00^*^
Psychological dimension	UA AIS UA × AIS	21.78 1.03 −0.70	7.40 0.15 0.22	0.00^*^ 0.00^*^ 0.00^*^
Appearance	UA AIS UA × AIS	4.10 0.44 −0.19	5.69 0.11 0.11	0.47 0.00^*^ 0.27
Personal relationship	UA AIS UA × AIS	18.34 0.58 −0.52	3.61 0.07 0.10	0.00^*^ 0.00^*^ 0.00^*^
AcroQoL All	UA Comorbidities UA × Comorbidities	−6.59 −4.31 1.61	5.97 1.17 1.99	0.27 0.00^*^ 0.42
Physical dimension	UA Comorbidities UA × Comorbidities	−1.39 −1.90 0.27	2.64 0.51 0.88	0.60 0.00^*^ 0.75
Psychological dimension	UA Comorbidities UA × Comorbidities	−5.52 −2.40 1.40	3.87 0.75 1.29	0.16 0.00^*^ 0.28
Appearance	UA Comorbidities UA × Comorbidities	−4.63 −1.27 0.72	2.52 0.49 0.84	0.07 0.01^*^ 0.39
Personal relationship	UA Comorbidities UA × Comorbidities	−0.88 −1.13 0.74	2.06 0.40 0.68	0.66 0.00^*^ 0.29
AcroQoL All	UA Symptoms UA × Symptoms	−20.47 −3.9 2.72	13.17 1.31 1.54	0.12 0.00^*^ 0.08
Physical dimension	UA Symptoms UA × Symptoms	−1.39 −1.90 0.27	2.64 0.51 0.88	0.60 0.00^*^ 0.75
Psychological dimension	UA Symptoms UA × Symptoms	−5.52 −2.40 1.40	3.87 0.75 1.29	0.16 0.00^*^ 0.28
Appearance	UA Symptoms UA × Symptoms	−4.63 −1.27 0.72	2.52 0.49 0.84	0.07 0.01^*^ 0.39
Personal relationship	UA Symptoms UA × Symptoms	−0.88 −1.13 0.72	2.06 0.40 0.68	0.66 0.00^*^ 0.29

* *p < 0.05, statistically significant*.

Additionally, the interaction of the relationship between these two variables (AIS and disease activity) is statistically significantly different in the context of social relations in WHOQoL questionnaires. In the group of participants with controlled acromegaly, the higher the level of acceptance of the disease, the higher scores in social relations were observed. And in the group of participants with uncontrolled acromegaly, the relationship was negatively correlated, but the size effect was much smaller (*r* = 0.7178 vs. *r* = −0.1528, respectively). What is more, the level of acceptance of the disease affected environmental domain, regardless the disease activity (CA and UA) (*r* = 0.543 vs. *r* = 0.120) (Table 3).

No difference in acromegaly symptoms as well as in number of comorbidities were found between CA and UA but these two parameters affected the results QoL scores in AcroQol questionnaires and their domains, regardless the disease activity. The number of comorbidities, not disease activity, was important for the results in AcroTotal (CA vs. UA (*r* = −0.54 vs. *r* = −0.40, respectively) as well as for its domains (AcroPhy: *r* = −0.54 vs. *r* = −0.53; AcroPsy: *r* = −0.50 vs. *r* = −0.12; AcroApp: *r* = −0.013 vs. *r* = −0.43; AcroRel: *r* = −0.44 vs. *r* = −0.18, respectively). Similarly, symptoms of acromegaly contributed the results in AcroTotal (CA vs. UAG (*r* = −0.46 vs. *r* = −0.37, respectively) and its three domains (AcroPhy: *r* = −0.39 vs. *r* = −0.25; AcroPsy: *r* = −0.47 vs. *r* = −0.33; AcroRel: *r* = −0.40 vs. *r* = −0.42, respectively), independently of disease activity (Table 3).

Besides, the prevalence of psychopathological symptoms (GHQ-28) contributed the level of acceptance of the disease, regardless the disease activity (CA vs. UA) (*r* = −0.55 vs. *r* = −0.30, respectively). Similarly, somatic symptoms (GHQ-A), anxiety and insomnia (GHQ-B) and social dysfunction (GHQ-C) affected the level of acceptance of the disease, independently of disease activity (CA vs. AU) (*r* = −0.53 vs. *r* = −0.20; *r* = −0.55 vs. *r* = −0.31; *r* = −0.52 vs. *r* = −0.21, respectively). What is more, the duration of illness had an impact on AIS, irrespective of the disease activity (*r* = −0.47, *r* = −0.21, respectively). The AIS seems to be not affected by participants' age, number of comorbidities and symptoms (Table 4).

**Table 4:** Linear mixed model analysis—interaction effect between AcroQoL scores and AIS with GHQ-28 scores and duration of the disease in clinical groups.

**Tool**	**Interaction description**	**Estimate**	**Std error**	***p*-value**
AcroQoL All	UA GHQ-28 UA × GHQ-28	−6.36 −1.76 1.47	4.84 0.42 0.60	0.19 0.00^*^ 0.01^*^
Physical dimension	UA GHQ-28 UA × GHQ-28	−2.13 −0.81 0.65	2.15 0.18 0.27	0.33 0.00^*^ 0.02^*^
Psychological dimension	UA GHQ-28 UA × GHQ-28	−4.39 −0.94 0.81	3.14 0.27 0.39	0.16 0.00^*^ 0.04^*^
Personal relationship	UA GHQ-28 UA × GHQ-28	−0.83 −0.48 0.49	1.64 0.14 0.20	0.61 0.00^*^ 0.02^*^
AcroQoL All	UA GHQ-A UA × GHQ-A	−5.27 −4.82 4.09	4.49 1.21 1.69	0.24 0.00^*^ 0.01^*^
Physical dimension	UA GHQ-A UA × GHQ-A	−1.54 −2.14 1.74	2.03 0.54 0.76	0.45 0.00^*^ 0.02^*^
Psychological dimension	UA GHQ-A UA × GHQ-A	−3.87 −2.67 2.33	2.88 0.77 1.08	0.18 0.00^*^ 0.03^*^
Personal relationship	UA GHQ-A UA × GHQ-A	−0.35 −1.36 1.33	1.50 0.40 0.56	0.80 0.00^*^ 0.02^*^
AcroQoL All	UA GHQ-B UA × GHQ-B	−6.14 −4.87 3.45	5.18 1.22 1.75	0.24 0.00^*^ 0.05
Physical dimension	UA GHQ-B UA × GHQ-B	−2.55 −2.32 1.76	2.30 0.54 0.77	0.27 0.00^*^ 0.02^*^
Psychological dimension	UA GHQ-B UA × GHQ-B	−3.66 −2.55 1.62	3.37 0.79 1.14	0.28 0.00^*^ 0.15
Appearance	UA GHQ-B UA × GHQ-B	−2.22 −1.09 0.18	2.20 0.51 0.74	0.31 0.03^*^ 0.80
Personal relationship	UA GHQ-B UA × GHQ-B	−1.58 −1.44 1.56	1.72 0.40 0.58	0.36 0.00^*^ 0.01^*^
AcroQoL All	UA GHQ-C UA × GHQ-C	−5.29 −5.71 5.72	4.44 1.58 2.25	0.23 0.00^*^ 0.01^*^
Physical dimension	UA GHQ-C UA × GHQ-C	−1.79 −2.71 2.62	1.97 0.70 1.00	0.36 0.00^*^ 0.01
Psychological dimension	UA GHQ-C UA × GHQ-C	−3.69 −2.99 3.11	2.86 1.02 1.45	0.20 0.00^*^ 0.03
Personal relationship	UA GHQ-C UA × GHQ-C	−3.78 −1.65 1.70	1.84 0.65 0.93	0.04^*^ 0.01^*^ 0.07
AIS	UA GHQ-28 UA × GHQ-28	−1.06 −0.77 0.37	2.87 0.25 0.35	0.71 0.00^*^ 0.30
AIS	UA GHQ-A UA × GHQ-A	−1.44 −2.10 1.38	2.68 0.72 1.01	0.59 0.00^*^ 0.18
AIS	UA GHQ-B UA × GHQ-B	−1.41 −2.25 1.01	3.05 0.71 1.03	0.64 0.00^*^ 0.33
AIS	UA GHQ-C UA × GHQ-B	−1.03 −2.62 1.42	2.57 0.91 1.30	0.68 0.00^*^ 0.28
AIS	UA Duration of disease UA × Duration of disease	−3.52 −0.49 0.31	3.13 0.17 0.25	0.26 0.00^*^ 0.22

* *p < 0.05, statistically significant*.

In addition, the interaction of the relationship between the prevalence of all psychopathological symptoms (GHQ-28) and disease activity was tested and a statistically significantly difference in the context of QoL in AcroQoL questionnaires and its domains was observed in relation to the course of the disease. In both groups of participants, the higher prevalence of psychopathological symptoms (GHQ-28), the worse QoL scores was observed but in uncontrolled group the size effect was significantly smaller in AcroTotal (CA vs. AU) (*r* = −0.61, *r* = −0.16, respectively) as well as in AcroPhy (*r* = −0.63 vs. *r* = 0.432, respectively) and AcroPsy (*r* = −0.54 vs. *r* = −0.1, respectively). Moreover, in the subgroup of participants with controlled acromegaly, the more psychiatric distress, the lower scores in personal relationship domain was observed. And in the subgroup of participants with uncontrolled acromegaly, the relationship was positively correlated, but the size effect was much smaller (*r* = −0.51 vs. *r* = 0.01, respectively). What is more, the prevalence of psychopathological symptoms affected perception of appearance, irrespective of the disease activity (CA vs. UA) (*r* = −0.43 vs. *r* = −0.15). Otherwise, the interaction of the relationship between somatic symptoms (GHQ-A), anxiety and insomnia (GHQ-B) and social dysfunction (GHQ-C) with the disease activity was also a statistically significantly different in the context of QoL, in relation to the course of the disease. In the both analyzed groups the higher incidence of somatic symptoms, the worse QoL scores was observed in AcroTotal (CA vs. UA) (*r* = −0.59 vs. *r* = −0.14, respectively) as well as in AcroPhy (*r* = −0.58 vs. *r* = −0.18, respectively), AcroPsy (*r* = −0.54 vs. *r* = −0.1, respectively) and AcroRel (*r* = −0.31 vs. *r* = −0.02, respectively), but the size effect was significantly smaller in UA. Also, in the both group of participants, the more frequent occurrence of anxiety and insomnia, the lower QoL in AcroTotal (CA vs. UA) (*r* = −0.58 vs. *r* = −0.27, respectively) as well as in AcroPhy (*r* = −0.62 vs. *r* = −0.23, respectively) and AcroRel (*r* = −0.53 vs. *r* = −0.06, respectively) were observed and size effect again was significantly smaller in uncontrolled group. Further, the prevalence of anxiety and insomnia had an impact on psychological dimension (CA vs. UA) (*r* = −0.51 vs. *r* = −0.26, respectively) and appearance (*r* = −0.36 vs. *r* = −0.39, respectively), independently of disease activity.

Equally, in CA and in UA, the higher incidence of pathological symptoms in terms of social relation, the poorer QoL scores in physical domain were obtained (*r* = −0.58 vs. *r* = −0.03, respectively). Moreover, in the subgroup of participants with controlled acromegaly, the more psychiatric distress in terms of social relations, the lower scores in QoL was observed in AcroTotal and in AcroPsy. And in the subgroup of participants with uncontrolled acromegaly, the relationship was positively correlated, but the size effect was very weak and much smaller (CA vs. UA) (*r* = −0.54 vs. *r* = 0.02 and *r* = −0.47 vs. *r* = 0.02, respectively).

The prevalence of psychopathological symptoms affected environmental domain of the WHOQoL-BREF, regardless the disease activity (CA vs. UA) (*r* = −0.30 vs. *r* = −0.34). Additionally, the psychiatric distress in terms of social relation affected physical health as well as environmental domain, regardless the disease activity (CA vs. UA) (*r* = −0.36 vs. *r* = −0.28; *r* = −0.26 vs. *r* = −0.36, respectively). What is more and very interesting, depressive symptoms had an impact on environmental domain, irrespective of disease activity (*r* = −0.22 vs. *r* = −0.38, respectively) (Table 4). However, there was no relation between QoL in AcroQoL and incidence of depressive symptoms.

Moreover, the level of GH had an impact of QoL results in AcroQol, regardless disease activity. These influences were observed in scores of AcroTotal (CA vs. UA) (*r* = −0.13 vs. *r* = −0.54) as well as in AcroPsy (*r* = −0.12 vs. *r* = −0.51). Also, the level of GH affected the prevalence of somatic symptoms (GHQ-A), independently of disease activity (CA vs. UA) (*r* = 0.09 vs. *r* = 0.6). Similarly, the level of IGF-1 contributed the prevalence of psychopathological symptoms (GHQ-28) and psychiatric distress in relation of social dysfunction, irrespective of disease activity (CA vs. UA) (*r* = 0.07 vs. 0.48; *r* = 0.23 vs. 0.51, respectively). However, what should be pointed out in each of the described cases, size effect was larger in the group of uncontrolled acromegaly, despite the fact that this difference did not meet the criterion of statistical significance.

A multivariate linear regression with interaction term gathering the most important factors was calculated in order to find essential elements of QoL among patients living with acromegaly (Table 5). The strongest predictors of QoL were related to the level of illness acceptance (*p* = 0.01) as well as the growth hormone level in the serum (*p* = 0.04). Disease activity was close to statistical significance (*p* = 0.06) in the described model. The model explained 60% of the variance of health-related QoL measured with AcroQol.

**Table 5:** Multivariate linear regression model with interaction term of health-related QoL.

	**Estimate**	**Std error**	***p-*value**
AcroQol total (Intercept)	50.2357	18.0537	**0.009**
UCA	−8.6614	4.4240	0.06
Duration of the disease	−0.1219	0.2422	0.6
Age	−0.1588	0.1187	0.2
Comorbidities	−1.5082	0.9362	0.1
Symptoms	−0.6575	0.8209	0.4
GH	−0.4957	0.2341	**0.04**
IGF-1	0.0154	0.0096	0.1
GHQ-28	−0.4671	0.3640	0.2
AIS	9.8826	0.3320	**0.01**

Bold values indicate p < 0.05, statistically significant.

## Discussion

In this paper psychological factors of patients harboring acromegaly and assessment of their relationship with the perceived QoL in the context of controlled or uncontrolled course of the disease were analyzed. The connections between acromegaly and QoL are very complex and still a matter of debate. Optimization the disease control, a core ambition of medical interventions, contributes to restoring life expectancy, lowering morbidity rates as well as costs reduction (2) but successful treatment in the terms of establishing biochemical control is controversial (3, 16–19, 24). As recalled in a paper by Matta et al. it may have positive, neutral or even negative relation to patient's QoL (9). Some authors have not observed better AcroQoL outcomes in controlled group (24) and, surprisingly, proved higher scores in active period of disease (18).

In our research, growth hormone level in the serum turned out to be one of the predictors of self-perceived QoL regardless disease activity. However, the size effect was larger in the group of uncontrolled acromegaly, despite the fact that this difference did not meet the criterion of statistical significance. Observation on the importance of that particular hormone concentration is consistent with data published by (25) suggesting IGF-1 and GH control may mediate AcroQoL scores in different ways. According to that study duration of IGF-1 normalization was positively related to total and all subscale scores while duration of GH control affected positively total AcroQoL as well as appearance, personal relationships subscales (25). Notably, the need of GH lowering therapy in line with duration of biochemical control were described as the crucial aspects affecting patients' QoL (25).

It also seems fair to emphasize that no matter what treatment modality was used, 22 of 50 patients' biochemical profile in our study was assessed as controlled which is consistent with recent research as recalled in (3) that states than <50% is to achieve disease control understood as hormone levels normalization. In a line with mentioned growth hormone level, AIS emerged as fundamental factor affecting QoL. In both groups considered, our results indicated on moderate AIS. Interestingly, there was a trend to note higher scores in uncontrolled subgroup. The difference, however, was not significant. One of possible explanations of such observations could be patients' expectations and perceptions that as long as excessive hormone secretion, a biochemical stigma of acromegaly, is to be normalized, there is still hope that perceived symptoms attributed to the disease would be reduced. At the time when hormone levels control is obtained and disease manifestations are persistent, patient could have a sensation that all the management options were already made used of and feel confronted with their irreversibility of physical changes, a constant need for medical attention or illness perceptions and ineffective coping strategies (24). This, in turn, could result in worse acceptance of the illness and consequently worse QoL perception. Taking different point of view, as shown in our study, the more acceptance, the better QoL measured with AcroQoL and WHOQOL-BREF was recorded in both studied groups especially in environmental and social domains of the latter scale. However, it should be emphasized that the relationship between the level of acceptance of the disease and the QoL domains seemed to be more significant in the controlled acromegaly subgroup where the size effect of the correlations was much bigger. The importance of perception of the illness and emotional representation of symptoms that could result in the level of acceptance affects self-perceived QoL (24, 26) thus should not be ignored by health providers, rather treated as a starting point of conversation. However, biochemical normalization cannot be simply translated as lessening acromegaly burden of concomitant diseases, thus biochemical control does not equal clinical control which is more complex and multidimensional. This statement could be backed with our observation that controlled and uncontrolled groups did not differ in number of symptoms and comorbidities, but these factors turned out to alter AcroQoL outcomes. Moreover, higher number of symptoms attributed to acromegaly was parallel with lower outcomes of used QoL questionnaires. Additionally, the research showed similar trend when number of comorbidities is considered in both, controlled and uncontrolled, groups. Similar tendency was recognized in relation to presence psychopathological symptoms in controlled and uncontrolled acromegalic patients, where no remarkable differences were detected. However, higher prevalence of mental distress turned out to be connected with worse outcome in the terms of QoL but in uncontrolled group the size effect was significantly smaller. What is more, the worst disturbances were observed in anxiety and insomnia scale. Regardless of disease activity the presence of mentioned psychopathological manifestations affected environmental domain of WHOQoL-BREF, similarly to depressive symptoms in both controlled and uncontrolled subgroups. This suggests that the presence of depression could be a modifiable factor when pursuing better QoL in the terms of this questionnaire. Remarkably, our results suggest that depressive symptoms do not alter QoL measured with AcroQoL These observations somewhat contrast with the outcome of the research carried out by Geraedts et al. that proved that the presence and intensity of depressive and anxiety symptoms can remarkably predict QoL tested with AcroQoL in acromegalic patients and concluded that the bigger amount of psychopathology, the bigger impairment in QoL can be expected (27). Nonetheless, data suggesting no relation between depressive symptoms and evolution of QoL in time also can be found (27). Intercorrelation between acromegaly, presence of depressive symptoms and satisfaction of patients was also found by Kepicoglu et al. (16). This being said, psychopathology is suggested not only to be an independent factor modifying QoL (15, 26, 27), but also superior to biochemical control and other factors. Proper consideration of the role of psychopathology plays a major role in holistic attitude toward such patients. There is significant amount of factors that could also contribute to QoL of patients living with acromegaly such as age, duration of the disease, gender, and treatment modalities. Longer duration of the disease along with older age were more prevalent in an uncontrolled group in our research and are affirmed to be a negatively influencing considerations for the outcomes of used questionnaires. Additionally, the longer time of experiencing disease in patients with acromegaly correlated with the lower AIS in the both groups. Duration can be considered in a three-way manner—duration of the disease, duration of biochemical control, and duration of remission. Duration of biochemical control in the line with the need GH lowering therapy were described as the predominant factors negatively affecting patients' QoL (25). Interestingly, Vandeva et al. stated that longer duration of disease remission negatively affected personal relations score measured with AcroQoL with likelihood of worse total and psychological rates (13). Additionally, researchers suggest that QoL tend to decrease with time, with no significant role of biochemical control and age. Similar conclusions on personal relations scores could be found in series of papers (15, 28). Possible explanations for such outcome could be the presence of irreversible physical changes, a constant need for medical attention or illness perceptions and ineffective coping strategies in mentioned group (24). Kyriakakis et al. points out that impaired psychosocial well-being is secondary to diminished physical function (25). Moreover, older age may predict worse AcroQoL scores of all scales, in both controlled and uncontrolled groups, apart from appearance score in controlled patients (13). Additionally, according to mentioned paper, age had borderline influence as a predictor of better scores in physical scale when baseline and prospective group of patients were compared. Data on relations between gender of acromegalic patients and their QoL is not consistent. According to Psaras et al. both genders are affected by long-lasting consequences of acromegaly, but interestingly, comorbidities varied in frequency between males and females and affected them differently in matters of QoL (29). There could also be sex-related variations in perceptions as well as response to therapy (30). Meanwhile, in cross-sectional study carried out by Vandeva et al. active disease in men was related to better outcome of all scales when compared to woman (13). It needs to be mentioned that there is a body of data that claims contrary (15, 16, 31). In the paper by Kyriakakis et al. women were characterized by higher QoL measured with AcroQoL in physical subscale (25). However, mentioned female gender as negative independent predictor of QoL, especially when biochemical control was not obtained, in all scales apart from the appearance subscale (13). Our results showed that gender as well as treatment modalities did not affect the QoL. In our study 36 out of 50 patients underwent surgical intervention but only 10 did not require additional medical treatment after the operation. Literature data on the association of QoL and biochemical disease control in patients with acromegaly are unclear. Data on diverse influence of particular types of drugs used on HRQoL can be found but is still controversial (13). Matta et al. points out that in patients who underwent operation persistent pituitary hormones hypersecretion is characterized by lower IGF-1 scores and therefore better performance in psychological subscale appearance score than in uncontrolled patients treated medically (9). Ishikawa et al. claims that QoL where endoscopic transsphenoidal approach is hired, could be improved by 6 months post-operation (32). Similarly, Mangupli et al. observed a significant improvement in the AcroQoL scores after disease control with octreotide LAR (4). On the other hand, Hua et al. showed negative correlation between treatment with lanreotide-controlled patients and QoL (17). In 26 of 50 participants of our study pharmacological treatment was applied. A group of 6 patients that were operated on needed adjuvant radiotherapy sessions which could be caused by exceptionally aggressive course of the disease or treatment resistance. This treatment modality proved to have a seriously affect AcroQoL rates and result in worse QoL (13) which is explained by the risk of, among others, long-term neurocognitive impairment (33) or development of hypopituitarism (34). Nevertheless, such trend was not present in our study. In turn, lack of impairment of anterior pituitary axis is said to result in better scores in domain of the appearance and have borderline significance on the total AcroQoL improvement prospectively (13). Analysis of this study revealed no correlation between prior radiotherapy and QoL scores. Biermasz et al. observed that hypopituitarism was significantly more frequent when radiotherapy due to acromegaly was performed (15). Interestingly, T'Sjoen et al. pointed out that it was not radiotherapy itself but deficiency in at least one pituitary axis that affected significantly psychological dimension of the AcroQol (28). Similarly, hypopituitarism as a result of surgery was proved not affect QoL as measured with AcroQoL in both primary controlled and uncontrolled acromegalic patients in at least 3-month post-operation observation as long as proper hormone replacement therapy was introduced (9). However, patients requiring hormone replacement therapy perceived their treatment as less controlled (24). What is more important and was decline in previous studies the degree of hypopituitarism may play an important role in its association with QoL (27). However, it needs to be emphasized that AcroQoL may not be a suitable tool in mentioned group of patients (35).

## Conclusions

As hormones concentrations are well-known and recognized factors to monitor the course of disease, more attention should be paid to AIS and modalities that contribute to it. The difference in the perceptions of patients with controlled and uncontrolled acromegaly emerges as the most remarkable conclusion from this study. Even though no significant differences in the variables analyzed individually in our study were identified, situation changes as relationships between variables are taken into consideration. Minding people with uncontrolled acromegaly, the control of biochemical factors seemed to be more important for the QoL perception, while among patients with controlled acromegaly, psychological variables such as acceptance of the disease are observed to play a fundamental role in QoL. Moreover, inclusion of patient's acceptance of the illness into clinical routine would promote holistic, patient-centered care and empower doctor-patient partnership where patients' expectations and perceptions are constantly tracked. Obtaining biochemical control should not be considered as the only measure of treatment success. The presence of psychopathology also needs to be emphasized, in contrast to age, gender, or duration of the disease, is potentially modifiable and could be targeted with suitable treatment.

## Strengths and Limitations

Among strengths and limitations of this research, a few things deserve to be emphasized. Studied population could be considered as small from the perspective of cross-sectional study design. On the other hand, the numerousness of the group seems to be adequate and sufficient, when compared to current literature, minding the fact that acromegaly is a relatively rare disease. Moreover, the groups' sociodemographic profiles were homogeneous in the terms of gender, education, place of residence, and marital status but differed statistically significantly in terms of age and duration of the disease. Percentage of the patients who improved and used treatment modalities were similar when contrasted with (remaining) data found in the literature. One of the limitations of current paper may be the lack of a control group in the analyzes presented. However, it should be mentioned that such study design is not accidental. This paper was aimed at detailed comparative analyzes between two groups of patients living with acromegaly differing in disease activity, and not analyzing individual variables compared to the group of healthy participants who do not have to face the consequences resulting from illness or their level of acceptance. Results on the QoL between different clinical groups and healthy participants were presented in another paper in details (26).

## Practical Implications of the Study/Future Interests

To the best of our knowledge this is study to highlight the complex interaction between QoL concept and the acceptance of diseases phenomenon in relation to the biochemical course of acromegaly. There is more and more attention paid to the vital importance of self-perceived QoL in chronic diseases in research but keeping it at a certain level should be perceived as one of the crucial goals of the everyday therapy. Pursuing optimal comorbidity management as soon as possible as prophylaxis of their possible irreversible consequences, could result in better QoL in acromegaly patients. Minding psychiatric symptoms is not to be underappreciated as independent, potentially modifiable contributors to QoL. Noteworthy, total cost of management of uncontrolled acromegaly is said to be higher when contrasted with controlled patients and treatment of coexistent diseases could enhance this trend. There is a need to determine and describe links between given factors (serum levels, duration, age, gender, acceptance, etc.) to conclude which are crucial and could be monitored in order to improve QoL in clinical environment. The stress should be put on modifiable considerations. Moreover, we are also still lacking data on more “global level” to support relations between biochemical control, treatment and better QoL (27).

## Data Availability Statement

All datasets generated for this study are included in the article/supplementary material.

## Ethics Statement

This study was carried out in accordance with the recommendations of local Bioethics Committee Medical University, Wroclaw. The protocol was approved by the local Bioethics Committee of Medical University, Wroclaw, Poland. All subjects gave written informed consent in accordance with the Declaration of Helsinki.

## Author Contributions

AJ-P and DS designed the project, the main conceptual ideas and proof outline, conducted study, interpretation of the results, complied the literature sources, wrote manuscript, and checked the references. MC complied the literature sources, wrote manuscript, and reference checking. MB and JR contributed conception and design of the study, helped in the interpretation date, and reference checking. All authors contributed to the final version of the manuscript and approved it for publication.

### Conflict of Interest

The authors declare that the research was conducted in the absence of any commercial or financial relationships that could be construed as a potential conflict of interest.

## References

[1] Lavrentaki A Paluzzi A Wass JAH Karavitaki N. Epidemiology of acromegaly: review of population studies. Pituitary. (2017) 20:4–9. 10.1007/s11102-016-0754-x 27743174 PMC5334410

[2] Chanson P Salenave S Kamenicky P Cazabat L Young J. Pituitary tumours: acromegaly. Best Pract Res Clin Endocrinol Metab. (2009) 23:555–74. 10.1016/j.beem.2009.05.010 19945023

[3] Ben-Shlomo A Sheppard MC Stephens JM Pulgar S Melmed S. Clinical, quality of life, and economic value of acromegaly disease control. Pituitary. (2011) 14:284–94. 10.1007/s11102-011-0310-7 21597975 PMC3146976

[4] Mangupli R Camperos P Webb SM. Biochemical and quality of life responses to octreotide-LAR in acromegaly. Pituitary. (2014) 17:495–9. 10.1007/s11102-013-0533-x 24178448

[5] Webb SM Badia X. Quality of life in acromegaly. Neuroendocrinology. (2016) 103:106–11. 10.1159/000375451 25661974

[6] Melmed S Casanueva FF Klibanski A Bronstein MD Chanson P Lamberts SW. A consensus on the diagnosis and treatment of acromegaly complications. Pituitary. (2013) 16:294–302. 10.1007/s11102-012-0420-x 22903574 PMC3730092

[7] Adelman DT Liebert KJP Nachtigall LB Lamerson M Bakker B. Acromegaly: the disease, its impact on patients, and managing the burden of long-term treatment. Int J Gen Med. (2013) 6:31–8. 10.2147/IJGM.S38594 23359786 PMC3555549

[8] Liu S Adelman DT Xu Y Sisco J Begelman SM Webb SM. Patient-centered assessment on disease burden, quality of life, and treatment satisfaction associated with acromegaly. J Investig Med. (2018) 66:653–60. 10.1136/jim-2017-000570 29151042

[9] Matta MP Couture E Cazals L Vezzosi D Bennet A Caron P. Impaired quality of life of patients with acromegaly: control of GH/IGF-I excess improves psychological subscale appearance. Eur J Endocrinol. (2008) 158:305–10. 10.1530/EJE-07-0697 18299462

[10] Roerink SHPP Wagenmakers MAEM Wessels JF Sterenborg RBTM Smit JW Hermus ARMM. Persistent self-consciousness about facial appearance, measured with the Derriford appearance scale 59, in patients after long-term biochemical remission of acromegaly. Pituitary. (2015) 18:366–75. 10.1007/s11102-014-0583-8 24965695

[11] Siegel S Streetz-Van Der Werf C Schott JS Nolte K Karges W Kreitschmann-Andermahr I. Diagnostic delay is associated with psychosocial impairment in acromegaly. Pituitary. (2013) 16:507–14. 10.1007/s11102-012-0447-z 23179964

[12] Abreu A Tovar AP Castellanos R Valenzuela A Giraldo CM Pinedo AC. Challenges in the diagnosis and management of acromegaly: a focus on comorbidities. Pituitary. (2016) 19:448–57. 10.1007/s11102-016-0725-2 27279011 PMC4935749

[13] Vandeva S Yaneva M Natchev E Elenkova A Kalinov K Zacharieva S. Disease control and treatment modalities have impact on quality of life in acromegaly evaluated by Acromegaly Quality of Life (AcroQoL) Questionnaire. Endocrine. (2015) 49:774–82. 10.1007/s12020-014-0521-6 25561370

[14] Trepp R Everts R Stettler C Fischli S Allemann S Webb SM. Assessment of quality of life in patients with uncontrolled vs. controlled acromegaly using the acromegaly quality of life questionnaire (AcroQoL). Clin Endocrinol. (2005) 63:103–10. 10.1111/j.1365-2265.2005.02334.x 15963069

[15] Biermasz NR Van Thiel SW Pereira AM Hoftijzer HC Van Hemert AM Smit JWA. Decreased quality of life in patients with acromegaly despite long-term cure of growth hormone excess. J Clin Endocrinol Metab. (2004) 89:5369–76. 10.1210/jc.2004-0669 15531483

[16] Kepicoglu H Hatipoglu E Bulut I Darici E Hizli N Kadioglu P. Impact of treatment satisfaction on quality of life of patients with acromegaly. Pituitary. (2014) 17:557–63. 10.1007/s11102-013-0544-7 24337714

[17] Hua SC Yan YH Chang TC. Associations of remission status and lanreotide treatment with quality of life in patients with treated acromegaly. Eur J Endocrinol. (2006) 155:831–7. 10.1530/eje.1.02292 17132752

[18] Webb SM Badia X Surinach NL, Spanish AcroQoL Study Group. Validity and clinical applicability of the acromegaly quality of life questionnaire, AcroQoL: a 6-month prospective study. Eur J Endocrinol. (2006) 155:269–77. 10.1530/eje.1.02214 16868140

[19] Bolanowski M Ruchała M Zgliczynski W Kos-Kudła B Hubalewska-Dydejczyk A Lewinski A. Diagnostics and treatment of acromegaly — updated recommendations of the Polish Society of Endocrinology. Endokrynol Pol. (2019) 70:2–18. 10.5603/EP.a2018.0093 30843181

[20] Goldberg DP Hillier VF. A scaled version of the general health questionnaire. Psychol Med. (1979) 9:139–45. 424481 10.1017/s0033291700021644

[21] Makowska Z Merecz D Przydatność Kwestionariuszy Ogólnego Stanu Zdrowia: GHQ-12 i GHQ-28 D. Goldberga w diagnozowaniu zdrowia psychicznego osób pracujacych. Med Pr. (2000) 6:589–601. 11288687

[22] Felton BJ Revenson TA Hinrichsen GA. Stress and coping in the explanation of psychological adjustment among chronically ill adults. Soc Sci Med. (1984) 18:889–98. 6729517 10.1016/0277-9536(84)90158-8

[23] R Core Team. A Language and Environment for Statistical Computing. Vienna, Austria (2019) Available online at: https://www.R-project.org/

[24] Tiemensma J Kaptein AA Pereira AM Smit JWA Romijn JA Biermasz NR. Affected illness perceptions and the association with impaired quality of life in patients with long-term remission of acromegaly. J Clin Endocrinol Metab. (2011) 96:3550–8. 10.1210/jc.2011-1645 21917873

[25] Kyriakakis N Lynch J Gilbey SG Webb SM Murray RD. Impaired quality of life in patients with treated acromegaly despite long-term biochemically stable disease: results from a 5-years prospective study. Clin Endocrinol. (2017) 86:806–15. 10.1111/cen.13331 28316090

[26] Szcześniak D Jawiarczyk-Przybyłowska A Matusiak Ł Bolanowska A Maciaszek J Sieminska M. Is there any difference in acromegaly and other chronic disease in quality of life and psychiatric morbidity? Endokrynol Pol. (2017) 68:524–32. 10.5603/EP.a2017.0044 28879648

[27] Geraedts VJ Dimopoulou C Auer M Schopohl J Stalla GK Sievers C. Health outcomes in acromegaly: depression and anxiety are promising targets for improving reduced quality of life. Front Endocrinol. (2014) 5:229. 10.3389/fendo.2014.00229 25610427 PMC4285111

[28] T'Sjoen G Bex M Maiter D Velkeniers B Abs R. Health-related quality of life in acromegalic subjects: data from AcroBel, the Belgian Registry on acromegaly. Eur J Endocrinol. (2007) 157:411–7. 10.1530/EJE-07-0356 17893254

[29] Psaras T Honegger J Gallwitz B Milian M. Are there gender-specific differences concerning quality of life in treated acromegalic patients? Exp Clin Endocrinol Diabetes. (2011) 119:300–5. 10.1055/s-0030-1267912 21031340

[30] Arnold AP. Promoting the understanding of sex differences to enhance equity and excellence in biomedical science. Biol Sex Differ. (2010) 1:1. 10.1186/2042-6410-1-1 21208467 PMC3010102

[31] Rowles SV Prieto L Badia X Shalet SM Webb SM Trainer PJ. Quality of life (QOL) in patients with acromegaly is severely impaired: use of a novel measure of QOL: acromegaly quality of life questionnaire. J Clin Endocrinol Metab. (2005) 90:3337–41. 10.1210/jc.2004-1565 15755865

[32] Ishikawa T Takeuchi K Nagatani T Aimi Y Tanemura E Tambara M. Quality of life changes before and after transsphenoidal surgery for sellar and parasellar lesions. World Neurosurg. (2019) 122:e1202–10. 10.1016/j.wneu.2018.11.017 30447458

[33] Spiegler BJ Bouffet E Greenberg ML Rutka JT Mabbott DJ. Change in neurocognitive functioning after treatment with cranial radiation in childhood. J Clin Oncol. (2004) 22:706–13. 10.1200/JCO.2004.05.186 14966095

[34] Jenkins PJ Bates P Carson MN Stewart PM Wass JAH. Conventional pituitary irradiation is effective in lowering serum growth hormone and insulin-like growth factor-I in patients with acromegaly. J Clin Endocrinol Metab. (2006) 91:1239–45. 10.1210/jc.2005-1616 16403824

[35] Deijen JB Arwert LI Witlox J Drent ML. Differential effect sizes of growth hormone replacement on quality of life, well-being and health status in growth hormone deficient patients: a meta-analysis. Health Qual Life Outcomes. (2005) 3:63. 10.1186/1477-7525-3-63 16236167 PMC1277839

